# Phase II randomized, double-blind, placebo-controlled study of tivantinib in men with asymptomatic or minimally symptomatic metastatic castration-resistant prostate cancer (mCRPC)

**DOI:** 10.1007/s10637-018-0630-9

**Published:** 2018-08-07

**Authors:** Paul Monk, Glenn Liu, Walter M. Stadler, Susan Geyer, Ying Huang, John Wright, Miguel Villalona-Calero, James Wade, Russell Szmulewitz, Shilpa Gupta, Amir Mortazavi, Robert Dreicer, Roberto Pili, Nancy Dawson, Saby George, Jorge A. Garcia

**Affiliations:** 1 Ohio State University, A433b Starling-Loving Hall, 310 W. 10th ave, Columbus, OH 43082 USA; 2 University of Wisconsin Carbone Cancer Center, 1111 Highland Ave, Madison, WI 53705 USA; 3 University of Chicago, 5841 S Maryland Ave, Chicago, IL 60637 USA; 4 University of South Florida, 4202 E Fowler Ave, Tampa, FL 33620 USA; 5 Ohio State University, 320 W 10th Ave, Columbus, OH 43210-1280 USA; 6 National Cancer Institute, 9609 Medical Center Dr., MSC, Bethesda, MD 9739 USA; 7 Miami Cancer Institute, 8900 N Kendall Dr, Miami, FL 33176-2118 USA; 8 Cancer Care Specialists of Central Illinois, 210 W Mckinley Ave, Decatur, IL 62526 USA; 9 University of Chicago Medical Center, 5841 S Maryland Ave # MC2115, Chicago, IL 60637-1447 USA; 10 University of Minnesota, 420 Delaware St SE, Minneapolis, MN 55455-0341 USA; 11 University of Virginia School of Medicine, PO Box 800716, Charlottesville, VA 22908-0716 USA; 12 Indiana University, 535 Barnhill Drive, Indianapolis, IN 46202 USA; 13 Georgetown-Lombardi Comprehensive Cancer Center, 3800 Reservoir Rd NW, Washington, DC 20007-2113 USA; 14 Roswell Park Cancer Institute, 6 Symphony Cir, Orchard Park, NY 14127 USA; 15 Taussig Cancer Institute, 9500 Euclid Ave, Cleveland, OH 44195-0001 USA

**Keywords:** Tivantinib, Castration resistant, Prostate, Cancer

## Abstract

*Background* Tivantinib is a non-ATP competitive inhibitor of c-MET receptor tyrosine kinase that may have additional cytotoxic mechanisms including tubulin inhibition. Prostate cancer demonstrates higher c-MET expression as the disease progresses to more advanced stages and to a castration resistant state. *Methods* 80 patients (pts) with asymptomatic or minimally symptomatic mCRPC were assigned (2:1) to either tivantinib 360 mg PO BID or placebo (P). The primary endpoint was progression free survival (PFS). *Results* Of the 80 pts. enrolled, 78 (52 tivantinib, 26 P) received treatment and were evaluable. Median follow up is 8.9 months (range: 2.3 to 19.6 months). Patients treated with tivantinib had significantly better PFS vs. those treated with placebo (medians: 5.5 mo vs 3.7 mo, respectively; HR = 0.55, 95% CI: 0.33 to 0.90; *p* = 0.02). Grade 3 febrile neutropenia was seen in 1 patient on tivantinib while grade 3 and 4 neutropenia was recorded in 1 patient each on tivantinib and placebo. Grade 3 sinus bradycardia was recorded in two men on the tivantinib arm. *Conclusions* Tivantinib has mild toxicity and improved PFS in men with asymptomatic or minimally symptomatic mCRPC.

## Introduction

Metastatic castration resistant prostate cancer (mCRPC) is the lethal version of this common disease. Prostate cancer reaches this point through the combined events of metastasis and adaptation by the tumor to a low testosterone environment. The overall survival of men with mCRPC has improved over the past few years with the introduction of several different agents with non-overlapping mechanisms of action. [1–5] Despite this progress, further improvement is needed as men with mCRPC still invariably succumb to this disease.

## C-MET and prostate cancer

Hepatocyte growth factor (HGF) and its receptor N-methyl-N′-nitrosoguanidine human osteosarcoma transforming gene (MET) seem to play important roles in the metastatic process [6, 7] and its signaling is abnormal in a variety of malignancies [8]. Serum HGF levels are higher in metastatic prostate cancer than in localized tumors [9] and has been associated with poorer outcomes. [10] Xenograft and in vitro data reveal that MET expression increases following androgen deprivation suggesting an association with the development of castrate resistant disease. [11, 12]

## Tivantinib

Tivantinib (ARQ 197; ArQule, Burlington, MA; Daichi-Sankyo, Tokyo, Japan) is an orally available selective small molecule that inhibits MET receptor tyrosine kinase with a novel ATP independent binding (allosteric inhibitor) mechanism, leading to inhibition of cell proliferation and induction of apoptosis in MET-expressing cancer cells. [13] [14, 15] Tivantinib has been found to have additional properties and in some preclinical studies its anti-cancer properties were independent of the c-MET inhibition. [16] Together, these findings supported the hypothesis that tivantinib would have activity against mCRPC. We therefore performed a phase II randomized placebo controlled trial of tivantinib in men with asymptomatic or minimally symptomatic mCRPC.

## Patients and methods

### Eligibility criteria

Eligible men were required to have metastatic histologically confirmed prostate adenocarcinoma, castrate testosterone level (<50 ng/dL), to be asymptomatic or minimally symptomatic (no symptoms attributable to prostate cancer greater than Grade 1), ECOG ≤2, and PSA ≥ 2 ng/ml. Prior treatment with sipuleucel- T and abiraterone acetate were allowed. Prior chemotherapy was not allowed unless used in a perioperative setting and completed >6 months prior to enrollment. Progressive disease at study entry was required and defined as two successive rises in PSA separated at least by one week, appearance of two or more new lesions on bone scan, > 20% objective increase in size of target lesion. This is consistent with Prostate Cancer Working Group 2 guidelines (PCWG2) for trials in advanced prostate cancer. [17] Bone targeting agents such as zoledronic acid or denosumab were permitted provided patients began therapy prior to study entry. Normal organ and bone marrow function were required. Exclusion criteria included radiotherapy within 4 weeks, uncontrolled intercurrent illness, known brain metastasis, history of myocardial infarction or unstable angina within 6 months, history of severely impaired lung function, active liver disease, poorly controlled diabetes, or impairment of gastrointestinal function. Institutional review board approval was obtained for all study procedures at each participating site. Each patient provided written informed consent.

### Treatment plan

Participants were stratified based on prior treatment with abiraterone acetate and sipuleucel-T and randomly allocated at a ratio of 2:1 to receive tivantinib or placebo in a double-blind fashion. Patients received twice-daily dosing of 360 mg tivantinib by mouth or matched placebo. One cycle was 28 days. At the time of disease progression, the blind could be broken and those assigned to the placebo arm were allowed to cross over to tivantinib. At the time of the trial conduct, abiraterone acetate was approved only in the post-docetaxel setting, and neither enzalutamide nor radium223 were approved. Therefore, placebo in this clinical setting was felt to be appropriate.

### Efficacy outcome measures

We used PCWG2 guidelines to define disease progression which included need for palliative radiation or surgery, RECIST 1.1 defined progression, the appearance of ≥2 new bone lesions on Tc^99^MDP bone scan (with instructions for recognizing flare). Investigator determined clinical deterioration was also considered progression. Rising PSA levels alone while on study drug were not considered disease progression. Toxicity was evaluated using National Cancer Institute Common Toxicity Criteria (version 4.0).

### Pretreatment and follow-up evaluations

At baseline, participants underwent complete history, physical examination and laboratory testing. Baseline imaging was completed ≤4 weeks prior to start of treatment. Patients were evaluated every 4 weeks with repeat examination, safety assessment and standard laboratory testing. Whole body bone imaging, CT of abdomen/pelvis and chest X-ray were performed every 12 weeks or as needed for symptoms suggestive of disease progression.

### Statistical considerations

The primary endpoint in this trial was to compare the PFS of tivantinib vs placebo. This was defined as the time from study entry (start of blinded treatment) to the date of documented progression and/or death, censoring alive and progression-free patients at their last follow-up date. In this trial, the proposed sample size of 78 eligible and evaluable patients (26 in the placebo arm, 52 in the tivantinib arm) provided 90% power to detect an improvement from 3 months median PFS with placebo to a median PFS of at least 6 months with the tivantinib treatment, and a Type I error rate of 0.10 was assumed for this one-sided test. This sample size was based on a log-rank test calculation using the R statistical program (gsDesign package, R version 2.11.1).

Since this was a phase II trial with a direct comparison between the treatment arm and a placebo-control arm, we relaxed the Type I error constraint to 0.10. [18] Progression free survival curves based on observed data were constructed using the Kaplan-Meier method, and Cox proportional hazards model was used to estimate the hazard ratio of treatment vs. placebo. Adverse events as defined by NCI CTCAE v4.0 were summarized using descriptive statistics, where the maximum grade for each type of toxicity was recorded for each patient, and frequency tables were made to determine toxicity patterns.

## Results

### Patient characteristics

**Fig. 1:**
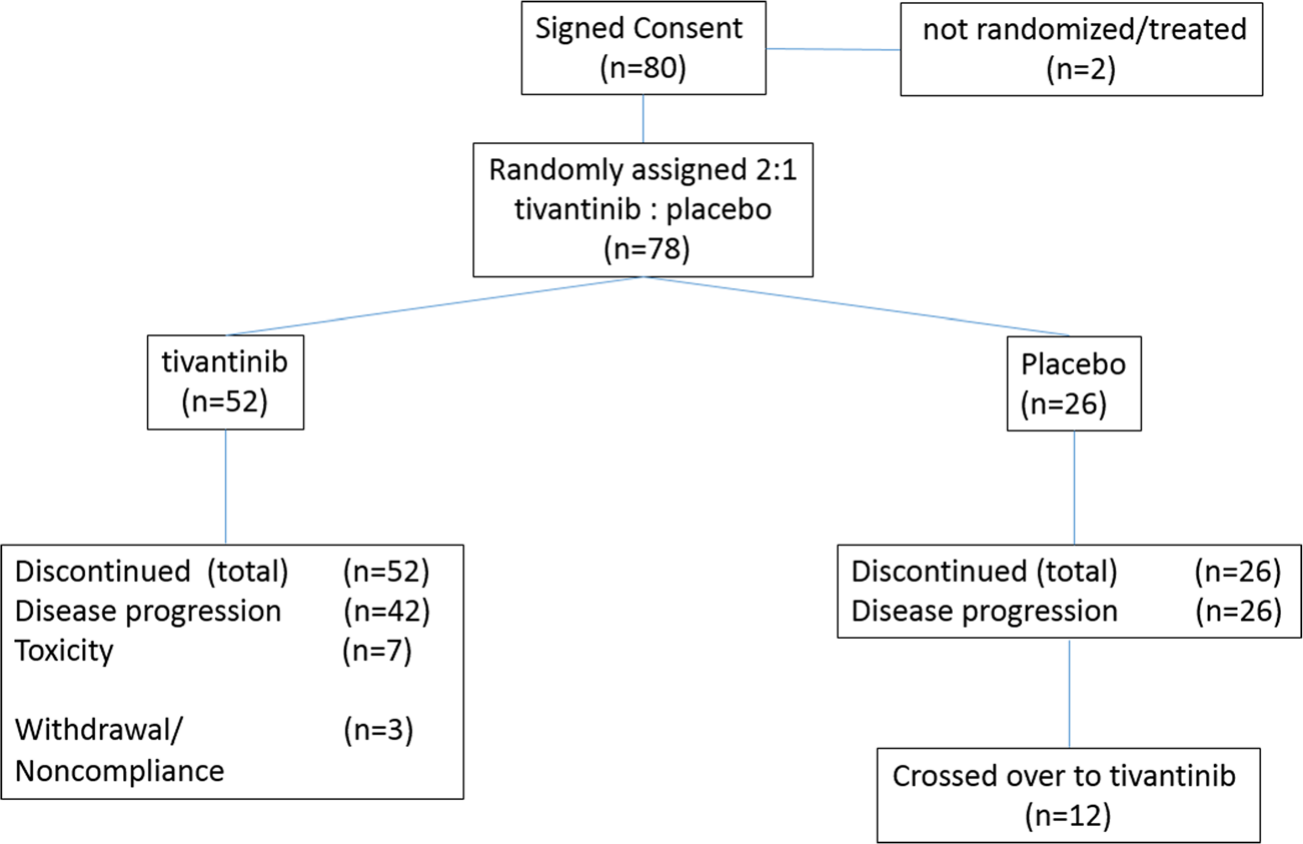
CONSORT diagram

Between January 2012 and September 2013 eighty men with asymptomatic or minimally symptomatic CRPC were enrolled in this multicenter, double-blind phase II trial. Seventy eight men (52 randomly assigned to tivantinib and 26 to matching placebo) started treatment and were included in safety and efficacy analysis. **(**Fig. 1**)** Groups were well balanced for most baseline characteristics **(**Table 1**)**. A higher proportion of men self-identifying as African American and men with lymph node involvement were randomized to placebo. There was no prior treatment with Radium-223, enzalutamide or chemotherapy while nearly a third of patients received prior abiraterone acetate and/or sipuleucel-T.

**Table 1:** Baseline Characteristics

Characteristic	Overall *N* = 78	Tivantinib *N* = 52	Placebo *N* = 26	P-value
Age at study entry Median (range)	67 (43–85)	67 (43–84)	66.5 (48–85)	0.93
Race				
Asian	1 (1%)	1 (2%)	0	0.015
African American	8 (10%)	2 (4%)	6 (23%)	
Caucasian	69 (88%	49 (94%)	20 (77%)	
Ethnicity				
non-Hispanic	75 (96%)	49 (94%)	26	0.55
unknown	3 (4%)	3 (6%)	0	
ECOG PS				
0	65 (83%)	42 (81%)	23 (88%)	0.53
1	13 (17%)	10 (19%)	3 (12%)	
Gleason Score				
< 7	9 (13%)	5(12%)	4 (16%)	0.25
7	17 25%)	10 (23%)	7 (29%)	
> 7	41 (61%)	28(65%)	13(54%)	
missing	11	9	2	
PSA				
median (range)	16.75 (2.2 to 868)	13.6 (2.3 to 868)	26.7 (2.2 to 579)	0.28
Alk phos				
median (range)	80 (16 to 423)	80.5 (41 to 423)	78 (16 to 322)	0.90
missing	1	0	1	
LDH				
median (range)	192 (111 to 770)	186 (126 to 770)	196 (111 to 467)	0.89
missing	5	4	1	
Hemoglobin				
median (range)	13 (10.1 to 38.9)	13.1 (10.6 to 38.9)	12.9 (10.1 to 14.5)	0.53
Bone involvement				
yes	50 (64%)	32 (62%)	18 (69%)	0.50
no	28 (36%)	20 (38%)	8 (31%)	
Lymph node involvement				
yes	17 (22%)	8 (15%)	9 (35%)	0.052
no	61 (78%)	44 (85%)	17 (65%)	
Lung involvement				
yes	5 (6%)	2 (4%)	3 (12%)	0.33
no	73 (94%)	50 (96%)	23 (88%)	
Other organ involvement*				
yes	12 (15%)	9 (17%)	3 (12%)	0.74
no	66 (85%)	43 (83%)	23 (88%)	
Prior Treatment				
Sipuleucel-T				
yes	24 (31%)	16 (31%)	8 (31%)	0.999
no	54 (69%)	36 (69%)	18 (69%)	
Abiraterone				
yes	23 (29%)	16 (31%)	7 (27%)	0.73
no	55 (71%)	36 (69%)	19 (73%)	

### Efficacy

**Fig. 2:**
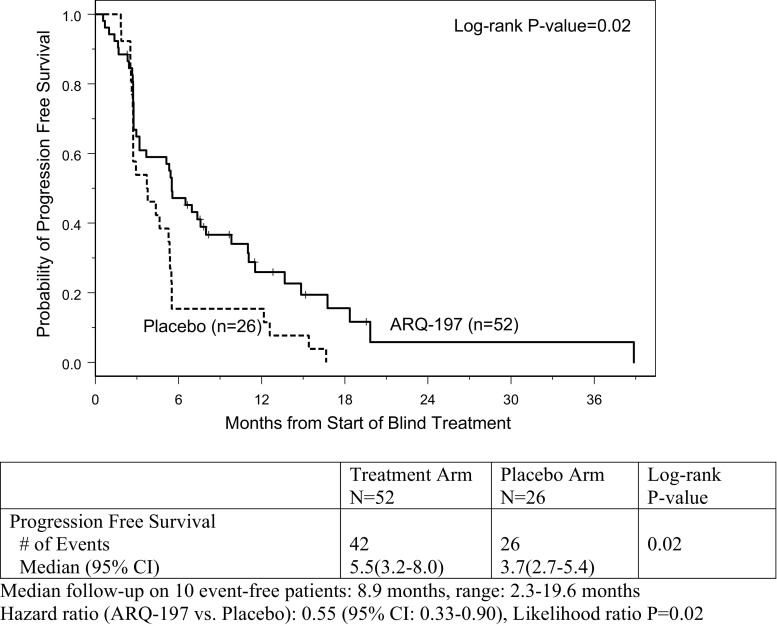
Progression Free Survival Curves

At the time of primary PFS analysis, 68 patients had progressed and/or died (26/26 on placebo and 42/52 on tivantinib). The median follow-up on event-free patients was 8.9 months (range: 2.3 to 19.6 months). The median PFS for those on the placebo arm was 3.7 months (95% CI: 2.7 to 5.4 months) vs. a median PFS of 5.5 months for those treated with tivantinib (95% CI: 3.2 to 8.0 months). **(**Fig. 2**).**

**Fig. 3:**
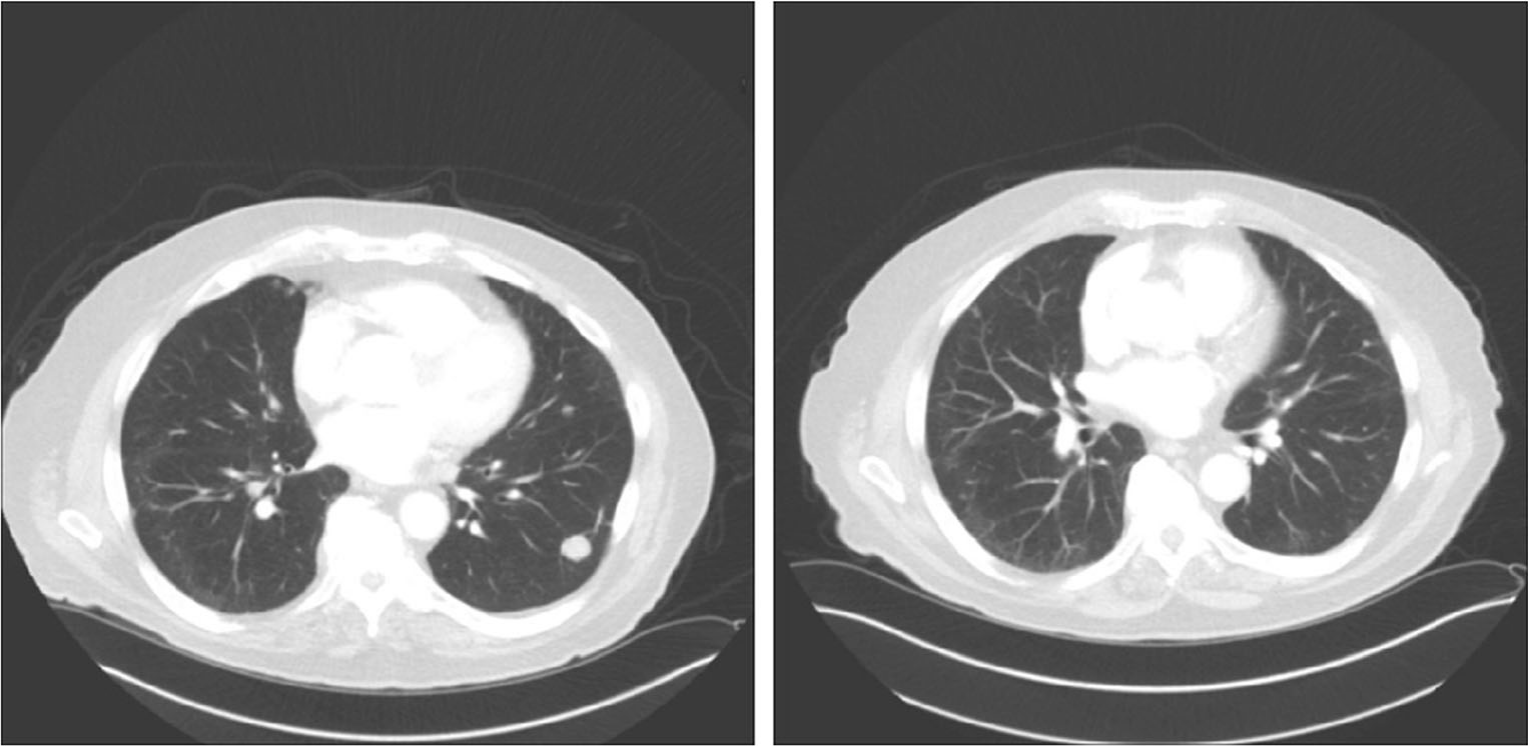
Pulmonary metastasis response to tivantinib

**Fig. 4:**
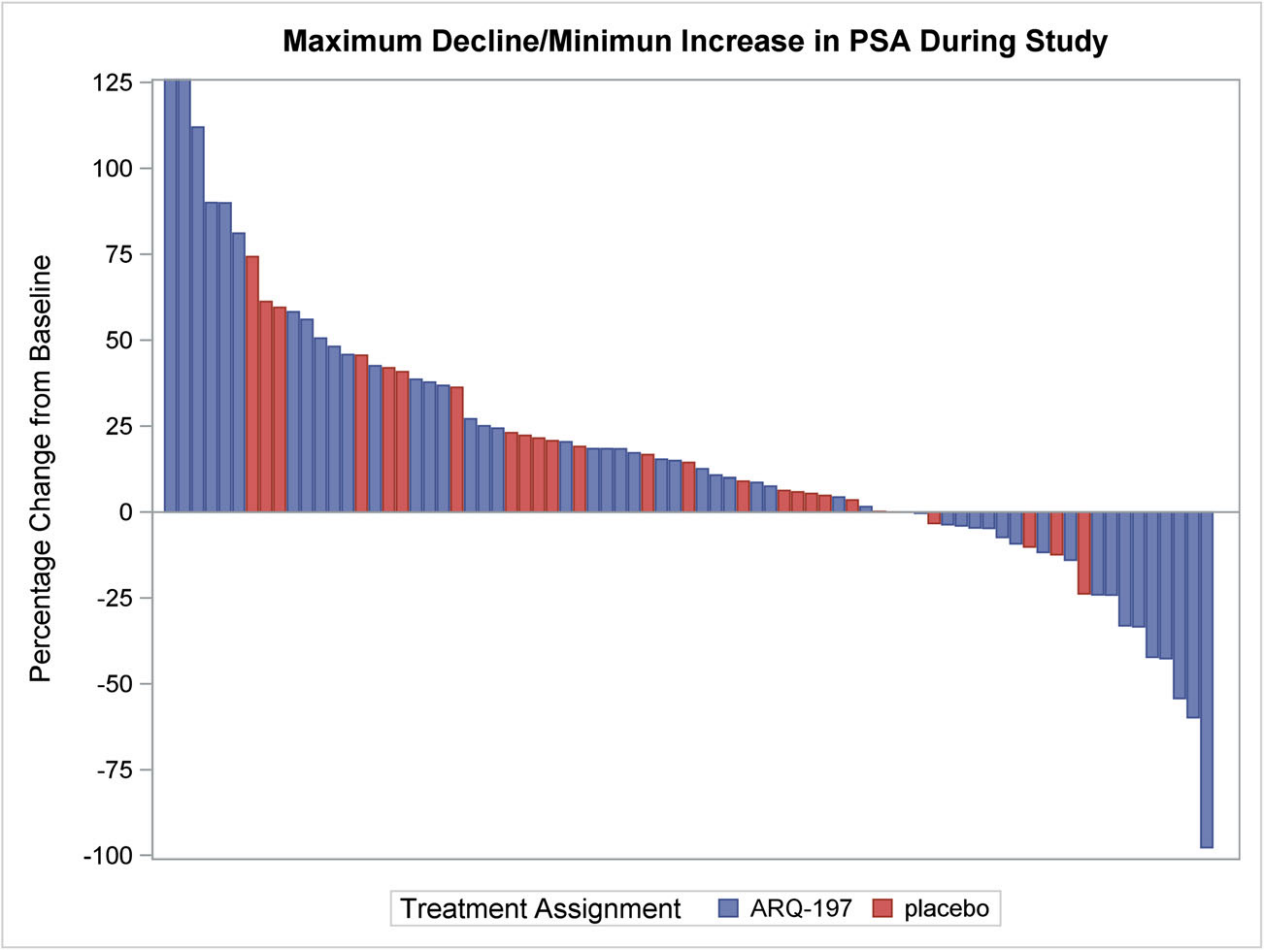
Maximum Decline/Minimum increase in PSA during study

A partial response by RECIST was documented in 1 patient randomized to tivantinib. **(**Fig. 3**)** Genomic profiling of this individual with an exceptional response revealed high androgen receptor amplification but no other significant alterations.PSA increases were generally seen on both arms. **(**Fig. 4**).**

Crossover from placebo to tivantinib was allowed at the time of progression. 12 of the 26 patients assigned to placebo when they progressed received tivantinib. The median time on tivantinib for this group was 4.3 months with a range of 2 to 10 months. One of the 12 experienced an objective partial response by RECIST. Overall survival was not measured.

### Safety

Toxicity is summarized in Table 2**.** Grade (G) 3 febrile neutropenia was seen in 1 patient on tivantinib while G3 and 4 neutropenia was recorded in 1 patient each on tivantinib and placebo. G3 sinus bradycardia was recorded in two men on the tivantinib arm. Eleven deaths (4 placebo and 7 tivantinib) were recorded during the trial and were all determined to be unrelated to therapy.

**Table 2:** Grade 3+ Adverse Events regardless of attribution

	*T (n = 52) *P (n = 26)	Grade 3/4	Grade 5
Acute coronary syndrome	T	1(2)	0(0)
	P	0(0)	0(0)
Back Pain	T	1(2)	0(0)
	P	2(8)	0(0)
Confusion	T	0(0)	0(0)
	P	2(8)	0(0)
Death NOS	T	1(2)	1(2)
	P	0(0)	1(4)
Dehydration	T	0(0)	0(0)
	P	3(12)	0(0)
Duodenal ulcer	T	1(2)	0(0)
	P	0(0)	0(0)
Dyspnea	T	1(2)	0(0)
	P	0(0)	0(0)
Dizziness	T	1(2)	0(0)
	P	0(0)	0(0)
Fall	T	0(0)	0(0)
	P	1(4)	0(0)
Fatigue	T	2(4)	0(0)
	P	0(0)	0(0)
Gait disturbance	T	0(0)	0(0)
	P	1(4)	0(0)
Generalized muscle weakness	T	0(0)	0(0)
	P	1(4)	0(0)
Hypertension	T	1(2)	0(0)
	P	0(0)	0(0)
Hypokalemia	T	0(0)	0(0)
	P	1(4)	0(0)
Hyponatremia	T	1(2)	0(0)
	P	0(0)	0(0)
Hypotension	T	0(0)	0(0)
	P	1(4)	0(0)
Hypoxia	T	1(2)	1(2)
	P	0(0)	0(0)
Infections	T	1(2)	0(0)
	P	3(12)	0(0)
Musculoskeletal and connective tissue disorder - Other	T	0(0)	0(0)
	P	1(4)	0(0)
Neoplasms benign, malignant and unspecified	T	0(0)	1(2)
	P	1(4)	0(0)
Nervous system disorders – Other	T	0(0)	0(0)
	P	1(4)	0(0)
Pleural effusion	T	0(0)	0(0)
	P	1(4)	0(0)
Sinus bradycardia	T	3(6)	0(0)
	P	0(0)	0(0)
Sinus Tachycardia	T	0(0)	0(0)
	P	1(4)	0(0)
Syncope	T	0(0)	0(0)
	P	2(8)	0(0)
Thromboembolic event	T	0(0)	0(0)
	P	1(4)	0(0)
Tumor Pain	T	1(2)	0(0)
	P	0(0)	0(0)
Urinary tract obstruction	T	0(0)	0(0)
	P	1(4)	0(0)
Hematologic AE, no (%)	Arm	Grade 3	Grade 4
Anemia	*T	3(6)	0(0)
	*P	2(8)	0(0)
Febrile neutropenia	T	1(2)	0(0)
	P	0(0)	0(0)
Neutrophil count decreased	T	1(2)	1(2)
	P	1(4)	1(4)
Platelet count decreased	T	1(2)	0(0)
	P	0(0)	0(0)
White blood cell decreased	T	1(2)	1(2)
	P	2(8)	0(0)

*T = tivantinib, P = placebo

## Discussion

Treatment with tivantinib was associated with minimal toxicity and a significantly longer PFS when compared to placebo in men with asymptomatic or minimally symptomatic mCRPC. In comparing the PFS distributions between treatment arms, the *p*-value for the log-rank test was *p* = 0.02. Furthermore, this p-value reflects a two-sided alternative hypothesis, which is more stringent than what was designed in this trial. Tivantinib’s favorable side effect profile has been demonstrated in various clinical trial settings, but these studies failed to achieve their respective primary endpoints. [19–23] This broad lack of efficacy is seen despite an underlying biologic rationale that is similar to the current trial. Several factors should be considered when interpreting the results of the present trial. First, the strengths of our report include the randomized design, the use of PCWG2 guidelines to determine progression and the control arm performed as expected. However, this study’s small size makes it more sensitive to biases that are potentially unaccounted for. More troublesome is the uncertainty of both the underlying mechanism of action of tivantinib and the value of PFS as an important endpoint in mCRPC trials. During the conduct of this trial, preclinical studies reported tivantinib’s activity is not via the inhibition of c-MET/HGF signaling. [16, 24] [25, 26] Rather, the in vitro activity is more consistent with a cytotoxic agent. Targeting MET therefore remains unproven as a strategy that produces clinical benefit in men with mCRPC. [27]

The inability to rely on intermediate endpoints to predict overall survival in mCRPC is problematic. [2, 28–30] This must be considered when we interpret the significant improvement in PFS seen in this study. The experience with cabozantinib’s development in mCRPC is perhaps most instructive. [31] Cabozantinib, a potent inhibitor of MET and VEGFR2, failed to improve overall survival (OS) when compared with prednisone in heavily treated men with mCRPC. These negative phase III results were accompanied by significant improvements in bone scan response, radiographic PFS, circulating tumor cell conversions, time to first symptomatic skeletal event and favorable bone biomarker changes. One potential explanation for the lack of OS benefit is the high number of dose reductions and discontinuations for toxicity. The phase II experience was associated with unprecedented tumor regression in the majority with soft tissue disease, normalization of bone scans in 12% and improvement in bone pain in 67%. [32] This apparent paradox seems most acute in advanced prostate cancer trials but it has been seen in other tumor types and caution has been advised when making conclusions with PFS data. [33]

## Conclusion

Tivantinib has mild toxicity and significantly improved PFS compared to placebo in men with asymptomatic/minimally symptomatic mCRPC. The magnitude of benefit does not support further evaluation as a single agent. Optimal further development of tivantinib in mCRPC would ideally include a better understanding of the drug’s underlying mechanism of action. This would better inform combination studies with other therapies in mCRPC.
